# The Relationships Between the Free-Living and Particle-Attached Bacterial Communities in Response to Elevated Eutrophication

**DOI:** 10.3389/fmicb.2020.00423

**Published:** 2020-03-25

**Authors:** Yang Hu, Guijuan Xie, Xingyu Jiang, Keqiang Shao, Xiangming Tang, Guang Gao

**Affiliations:** ^1^State Key Laboratory of Lake Science and Environment, Nanjing Institute of Geography and Limnology, Chinese Academy of Sciences, Nanjing, China; ^2^Nanjing Institute of Geography and Limnology, Chinese Academy of Sciences, Beijing, China

**Keywords:** free-living bacteria, particle-attached bacteria, suspended particles, carbon resource, metabolic functions

## Abstract

Exploring the relationships between free-living (FL) and particle-attached (PA) bacterial communities can provide insight into their connectivity and the partitioning of biogeochemical processes, which is crucial to understanding the elemental cycles and metabolic pathways in aquatic ecosystems. However, there is still intense debate about that whether FL and PA fractions have the same assemblage. To address this issue, we investigated the extent of similarity between FL and PA bacterial communities along the environmental gradients in Lake Wuli, China. Our results revealed that the west Lake Wuli was slightly eutrophic and the east lake was moderately and highly eutrophic. The alpha-diversity of the FL bacterial communities was significantly lower than that of the PA fraction in the west lake, whereas the alpha-diversity of the two fractions was comparable in the east lake. The beta-diversity of both communities significantly differed in the west lake, whereas it resembled that in the east lake. Moreover, functional prediction analysis highlighted the significantly larger differences of metabolic functions between the FL and PA fractions in the west lake than in the east lake. Suspended particles and carbon resource promote the similarity between the FL and PA fractions. Collectively, our result reveals a convergent succession of aquatic communities along the eutrophic gradient, highlighting that the connectivity between FL and PA bacterial communities is nutrient related.

## Introduction

Particles are crucial components in aquatic ecosystems (Simon et al., 2002), serving as pivotal energy sources for aquatic food webs and maintaining the nutrient budgets of carbon, nitrogen, and phosphorus (Sahoo et al., 2013). Because the particle microenvironment contains elevated substrate concentrations, particles are “hot spots” for bacterial growth and transformation processes (Tang et al., 2010; Ortega-Retuerta et al., 2013). According to their distance to particles, the bacteria are divided into two groups: free-living (FL) bacteria and particle-attached (PA) bacteria (Crump et al., 1998, 1999). The PA bacteria colonize and remineralize organic particles, releasing dissolved organic matter into the surrounding water, which provides important resource for their FL counterparts. Distinguishing between bacterial lifestyles can thus provide clues to understanding the elemental cycles and the metabolic pathways in aquatic ecosystems (Ortega-Retuerta et al., 2013).

Exploring the relationships between the FL and PA bacterial communities has been of long-standing interests to microbiologists. For decades, the FL and PA bacterial communities have been considered to be significantly different. The PA lifestyle has considerable advantages over the FL lifestyle, including access to resources and protection from predators and deleterious environmental pressures (Dang and Lovell, 2016; Liu et al., 2019). Furthermore, the higher amount of labile organic material in particles than in the surrounding water leads to the PA bacteria being mainly copiotrophic, whereas the FL bacteria are mainly oligotrophic (Koch et al., 2014; Dang and Lovell, 2016). In addition, properties of the particle microniche facilitate bacterial diversification, allowing the PA bacterial communities to be more diverse than the FL bacterial communities (Ortega-Retuerta et al., 2013; Tang et al., 2015). Taxonomically, the most abundant phylum of PA bacterial communities is affiliated with the Proteobacteria, whereas for the FL bacterial communities, the most abundant phylum belongs to the Actinobacteria (Suzuki et al., 2017; Yang et al., 2017). Thus, these results imply that, in aquatic ecosystems, the PA and FL bacterial communities are independent components of the aquatic bacterial assemblage.

However, many updated and novel findings show only slightly different FL and PA bacteria communities (Hollibaugh et al., 2000; Ortega-Retuerta et al., 2013; Dang and Lovell, 2016). Modern microbial ecology has shown that aquatic bacteria possess a complex lifestyle and frequently alternate between an FL stage and a PA stage (Grossart, 2010). The PA bacteria can also survive freely in the water column, so they can colonize new particles, and therefore, they contribute, at particular times, to the FL bacterial communities (Crespo et al., 2013). For instance, Selje and Simon (2003) reported similar dominant sub-communities between FL and PA fractions. Tang et al. (2017) also reported that 59% of operational taxonomic units (OTUs), which accounted for 96% of the whole reads shared by the FL and PA bacterial communities. Thus, these observations highlight a highly dispersal potential and connectivity between the FL and PA bacterial communities, so they may function as one ecological and biogeochemical unit with rapid exchange (Hollibaugh et al., 2000; Lamontagne and Holden, 2003; Tang et al., 2015).

Thus, any attempt to understand the aquatic communities must be placed in the context of the relationship between the FL and PA bacterial communities. Recent progress has found that the aquatic communities prefer an FL lifestyle when nutrient level is high, whereas they prefer a PA lifestyle when nutrient level is low (Dang and Lovell, 2016). Thus, we hypothesized the relationships between the FL and PA fractions are nutrient related. To verify this, we investigated the FL and PA bacterial communities along the elevated eutrophication in Lake Wuli, China. The following questions were addressed: (1) does the similarity between the FL and PA bacterial communities vary with different nutrient status? and (2) what are the factors that shape their relationships?

## Materials and Methods

### Study Area, Field Sampling, and Physiochemical Measurements

**FIGURE 1:**
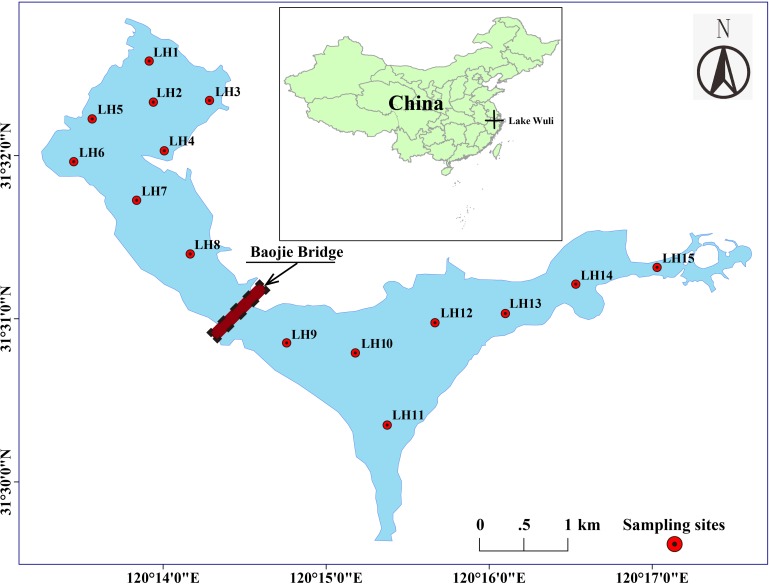
Location of the sampling sites in Lake Wuli.

Our studies were undertaken at Lake Wuli (120.22–120.29°E, 31.48–31.55°N) in the north of Lake Taihu, China. Lake Wuli has an area of 8.6 km^2^ (Wang et al., 2018) and is divided into two regions (the East Wuli Lake and the West Wuli Lake) by the boundaries of Baojie Bridge (Figure 1). Since the 1960s, Lake Wuli has experienced serious eutrophication accompanied by the occurrence of harmful algal blooms (HABs) (Li, 1996). Since 2002, the government has expended considerable effort to improve the water quality in Lake Wuli, with interventions such as sediment dredging, interruption of external nutrient input, and vegetation establishment. However, owing to industrial and sewage runoff through the river input in the east lake (Wang et al., 2019), most area of east lake is still characterized by *Microcystis* bloom. In contrast, the west lake flourished with aquatic plants, clear water, and abundant aquatic products (Gu and Lu, 2004). Being located in a subtropical monsoon climate zone, Wuli Lake has a prevailing southeast wind all year. Owing to the blocking effect of the Baojie Bridge, the east lake is more turbulent than the west lake (Wang et al., 2019).

In June 2017, field sampling was conducted at 15 sites, with eight sites in the west lake and seven sites in the east lake (Figure 1). At each site, surface water (top 50 cm) was collected with a 5 L Schindler sampler and then transported to the laboratory in a dark, cooled container. Although there is no standard definition of pore size to distinguish FL bacteria from PA ones, 3 and 5 μm of pore size have been the most widely applied in previous studies. During the current investigation, there were large particles of debris from *Microcystis* on the surface of Lake Wuli. In this case, 5 μm pore size is adequate to collect the PA bacteria (Tang et al., 2017; Liu et al., 2019). For each site, the PA bacteria were collected by filtering 400 ml of water through a 5 μm filter (Millipore, United States). The filtrate was then poured through a 0.22 μm filter to collect the FL bacteria. A total of 30 samples were collected (15 for FL bacteria and 15 for PA bacteria). All filters were stored at −80°C until nucleic acid extractions. The remaining subsample was held at 4°C for an immediate chemical analysis.

Secchi depth (SD) was determined by using a Secchi disk. Water temperature (Temp) and dissolved oxygen (DO) were assessed *in situ* by a multi-parameter water quality sonde (YSI 6600v2; United States). Eight additional characteristics were measured by standard methods (Jin and Tu, 1990): total nitrogen (TN), dissolved TN (TDN), total phosphorus (TP), dissolved TP (TDP), total suspended solids (TSS), chemical oxygen demand (COD), dissolved organic carbon (DOC), and chlorophyll *a* (Chl-*a*).

### DNA Extraction, PCR Amplification, and Illumina Sequencing

Total DNA from filtered bacterial community was extracted according to Zhou et al. (1996). Crude DNA extracts were then purified by the E.Z.N.A^®^ cycle-Pure kit (Omega Bio-Tek). The V4 regions of the 16S rRNA genes were amplified using the primers 515F (GTGYCAGCMGCCGCGGTAA) and 806R (GGACTACNVGGGTWTCTAAT) (Sáenz et al., 2019). Polymerase chain reaction (PCR) amplification was performed in a 50 μl reaction mixture containing 5 μl of 10× PCR buffer, 4 μl of MgCl_2_ (25 mmol/L), 0.5 μl of each primer (10 μmol/L each), 30 ng of quantified template DNA measuring by Pico green, and 0.4 μl of *Taq* polymerase (5 U/μl; Fermentas). To increase specificity and sensitivity during gene amplification, the touchdown PCR was conducted in a thermocycler (Applied Biosystems Veriti Thermal Cycler, United States): denaturation at 94°C for 5 min, 11 cycles of denaturation at 94°C for 1 min, annealing at 65°C for 1 min (temperature was decreased by 1°C every cycle until 55°C was reached), and extension at 72°C for 1 min (Don et al., 1991; Moezi et al., 2019). Nineteen additional cycles were performed at an annealing temperature of 55°C, followed by a final extension at 72°C for 10 min.

The pair-end sequencing was performed on an Illumina MiSeq platform. Unique barcodes were added to each sample, and the individual paired reads were initially merged by using FLASH (Fast Length Adjustment of Short reads) software (Magoč and Salzberg, 2011). We removed sequences that contained more than one ambiguous nucleotide that did not have a complete barcode and primer at one end or that were shorter than 200 bp after removal of the barcode and primer sequences. Chimeras were identified and were removed with the program usearch (version 10) (Edgar, 2010). OTUs were clustered with 97% similarity cutoff using vsearch (version 2.8.1) (Jackson et al., 2016). The representative sequence of each phylotype was selected and aligned by PDP Classifier (version 16) against the SILVA 16s rRNA database (release 132) with a confidence threshold of 70%. Sequences that belonged to Cyanobacteria were discarded from the subsequent analyses because this study focused on the heterotrophic bacterial communities only. Additionally, low confidence singletons (OTUs with a sequence count of smaller than 2) were excluded from downstream analyses.

### Data Deposition

The raw sequence data reported in this paper have been deposited in the Genome Sequence Archive (Genomics, Proteomics, and Bioinformatics 2017) in BIG Data Center (Nucleic Acids Res 2019), Beijing Institute of Genomics (BIG), Chinese Academy of Sciences, under accession number CRA002048, which is publicly accessible at https://bigd.big.ac.cn/gsa.

### Data Analysis

All statistical analyses and visualization were carried out using ape, factoextra, gglot2, Hmics, and vegan packages in the R environment (version 3.2.2)^1^.

As an indicator for assessing the trophic level according to the national standard, the trophic level index (TLI) is presented (Wang et al., 2002). The TLI value ranges from 0 to 100, with high values representing high levels of eutrophication. Trophic level is categorized into five grades: oligotrophic (TLI < 30), mesotrophic (30 ≤ TLI ≤ 50), slightly eutrophic (50 < TLI ≤ 60), moderately eutrophic (60 < TLI ≤ 70), and hyper-eutrophic (TLI > 70). The cluster analyses of Lake Wuli based on the environmental parameters were conducted by the *kmeans* function and were visualized by the *fviz_cluster* function.

Prior to bioinformatic analyses, the 16S OTU data were rarefied on the basis of the least reads across all samples. Alpha-diversity and beta-diversity of the bacterial communities were estimated by *alpha_div* and *beta_div* workflow according to usearch. We calculated four indices: Chao1, Richness, Simpson, and Shannon. To compare the statistical differences, we performed non-parametric Kruskal–Wallis rank tests. We also used non-metric multidimensional scaling (NMDS) analysis on the basis of four distance matrices: Bray–Curtis distance, Jaccard dissimilarity, and unweighted and weighted UniFrac distance. To determine significant difference of beta-diversity, ADONIS was used. To predict the functional profiles of bacterial communities, on the basis of gene bacterial community, we applied the Phylogenetic Investigation of Communities by Reconstruction of Unobserved States (PICRUSt) (Langille et al., 2013). To perform multiple comparisons of the relative abundance of functional gene categories and OTU abundance, we used STAMP (version 2.0.9). The correlation test between environmental parameters and the dissimilarity of FL and PA bacterial communities was conducted by the *rcorr* function.

## Results

### Characteristics of the Physicochemical Environment in Lake Wuli

**FIGURE 2:**
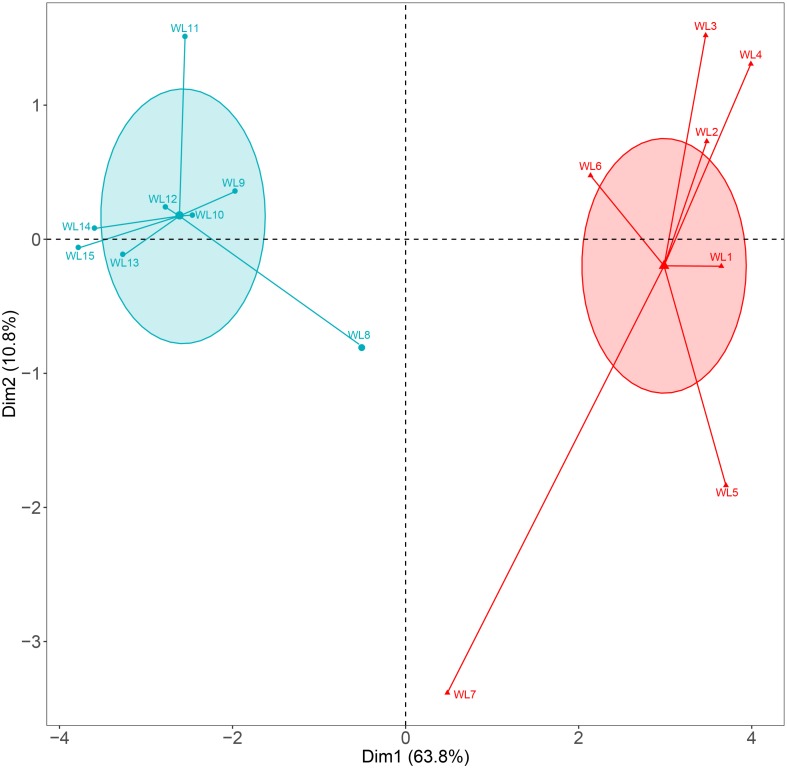
Cluster analysis of Lake Wuli based on the physicochemical parameters.

**FIGURE 3:**
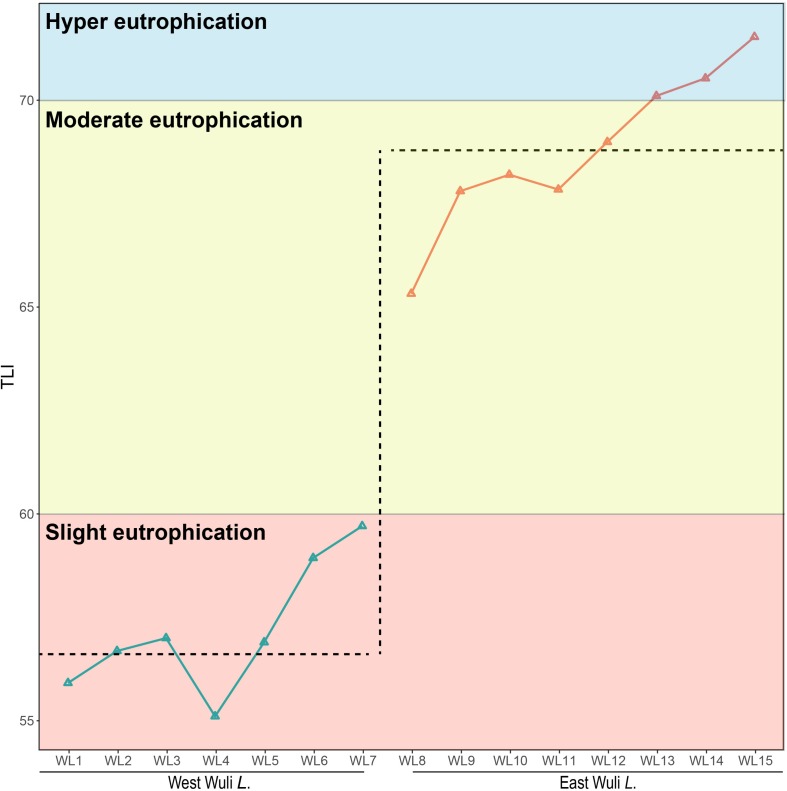
The eutrophic status of Lake Wuli.

Our results showed that the west lake and the east lake were distinct aquatic environments with statistically significant differences. Cluster analysis revealed that samples within the west lake and the east lake were separated from each other (Figure 2). We used the TLI as an index for accessing the eutrophication status of lake. In the west lake, the mean TLI was 56.61 ± 1.15, with which all eight sampling sites slightly eutrophic. By contrast, in the east lake, the mean TLI was 68.79 ± 1.60, within which sites WL8–WL12 and WL13–WL15 were moderately eutrophic and hyper-eutrophic, respectively (Figure 3).

The concentration of Chl*-a* was significantly lower in the west lake than in the east lake (64.00 ± 25.96 vs. 137.28 ± 36.51 μg/L; *P* < 0.001). The SD significantly decreased from 0.78 ± 0.06 m in the west lake to 0.29 ± 0.12 m in the east lake, whereas the TSS significantly increased from 14.83 ± 3.70 to 27.38 ± 4.64 mg/L (both *P* = 0.002). Water in the west lake contained more oxygen than that in the east lake (7.37 ± 0.60 vs. 6.13 ± 0.61 mg/L, *P* = 0.005). Of particular note was that the concentration of six nutrients significantly increased from the west lake to the east lake, namely, TN, TP, TDN, TDP, COD, and DOC (all *P* < 0.01).

### Convergent Succession Between the Free-Living and Particle-Attached Bacterial Communities From West Lake Wuli to East Lake Wuli

**FIGURE 4:**
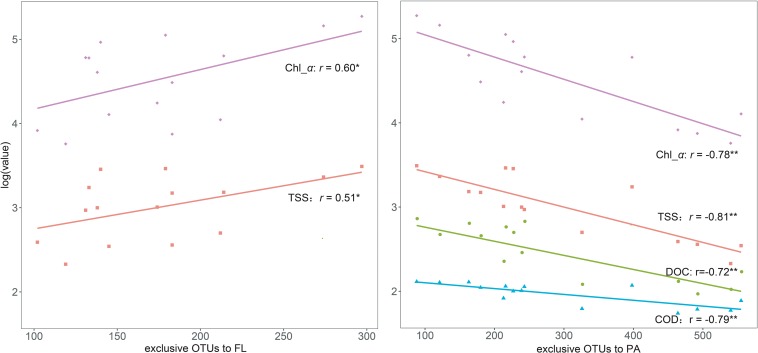
Linear relationships between the exclusive operational taxonomic units (OTUs) to free-living (FL) and particle-attached (PA) communities and the key aquatic environments.

In total, 159,054 high-quality sequences with 226 bp were generated among all sites after trimming, quality control, and singleton removal. Good’s coverage was 99.78–99.85%, suggesting that the sequencing effort was sufficient to capture the community diversity. This implication was also supported by the rarefaction curves, which approached asymptotes (Supplementary Figure S1). A total of 1,789 belonged to the FL bacterial community, and 1,629 OTUs belonged to the PA bacterial community. The number of OTUs exclusive to the FL bacteria increased from the west lake to the east lake, with significantly positive correlations with TSS and Chl-*a*. In contrast, the number of OTUs exclusive to the PA bacteria decreased from the west lake to the east lake, with significantly negative correlations with TSS, DOC, COD, and Chl-*a* (Figure 4).

**FIGURE 5:**
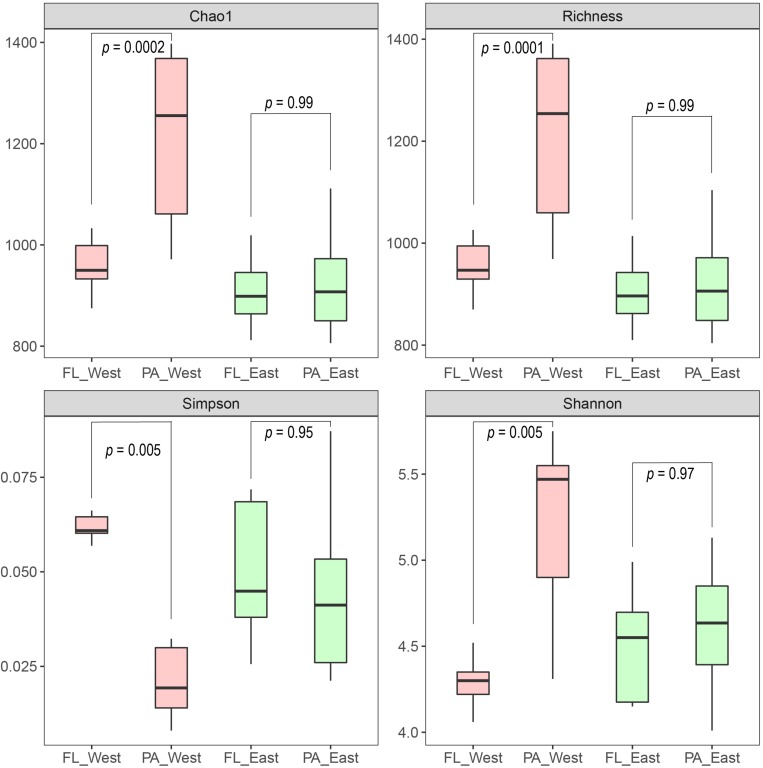
The alpha-diversity between the free-living (FL) and particle-attached (PA) bacterial communities within the west Lake Wuli and the east Lake Wuli.

**FIGURE 6:**
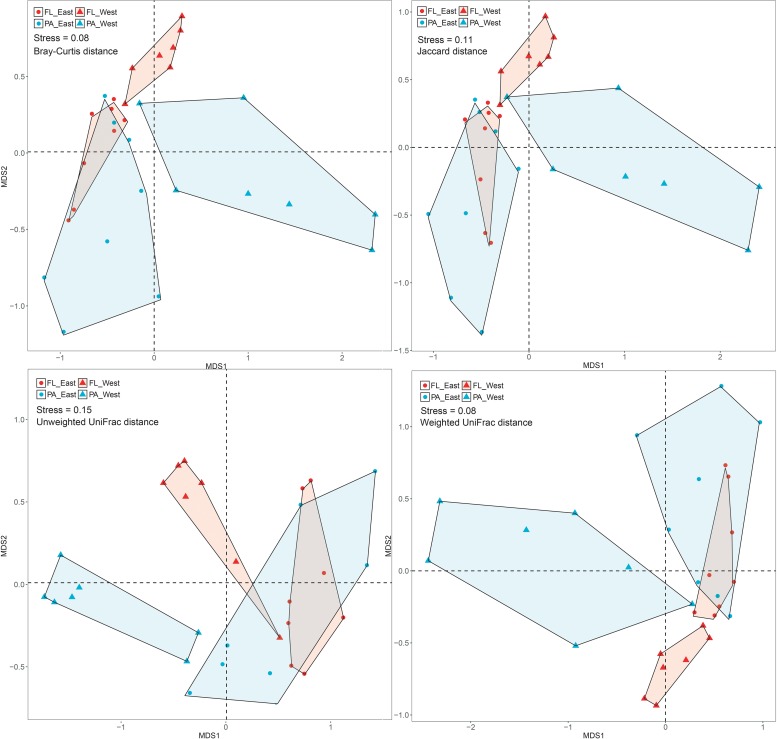
Non-metric multidimensional scaling analysis of the free-living (FL) and particle-attached (PA) bacterial communities in Lake Wuli.

**FIGURE 7:**
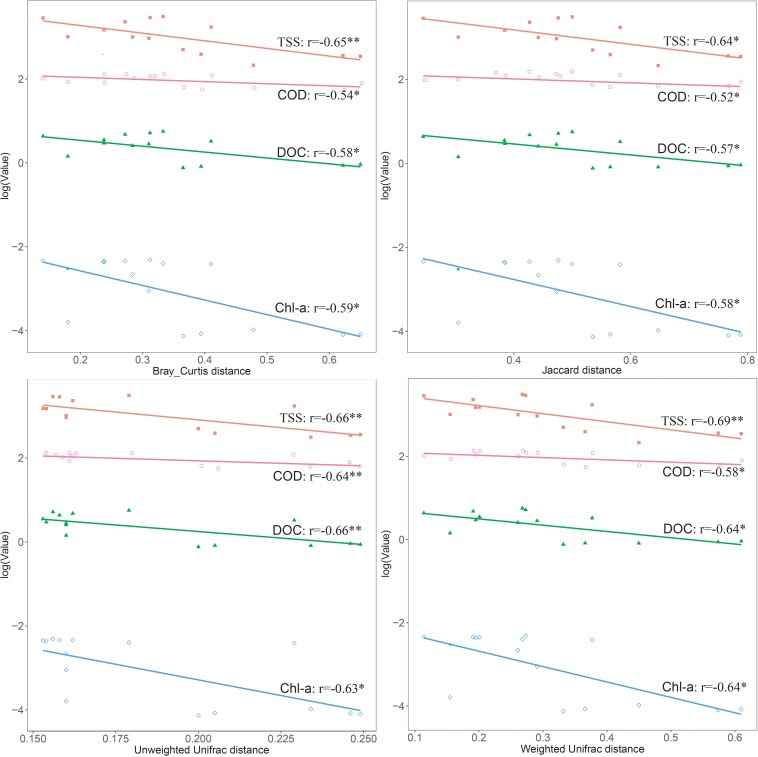
Linear relationships between the dissimilarity of the free-living (FL) and particle-attached (PA) bacterial communities and the physicochemical parameters in Lake Wuli.

After the sequencing depth to the least number of reads among each site (68,292 reads) was normalized, four indices were chosen to represent the community alpha-diversity, namely, Chao1, Richness, Simpson, and Shannon. In the west lake, the FL fraction was significantly less diverse than the PA fraction (all *P* < 0.01), however, in the east lake, the FL and PA fractions were as diverse as each other (all *P* > 0.05) (Figure 5). The beta-diversity of the FL and PA fractions was also evaluated according to a NMDS plot (Figure 6). The results showed that in the west lake, there was a clear segregation between them; this was also confirmed by the Adonis test (all *P* < 0.01). In contrast, in the east lake, the difference between FL and PA fractions was insignificant (all *P* > 0.05). Together, the FL and PA bacterial communities exhibited a convergent succession from the west to east lake. To detect the potential factors influencing the similarity between these two communities, we used the linear fitting approach. High and significantly negative associations are found between their similarities and DOC, COD, TSS, and Chl-*a* (Figure 7, all *P* < 0.05).

**FIGURE 8:**
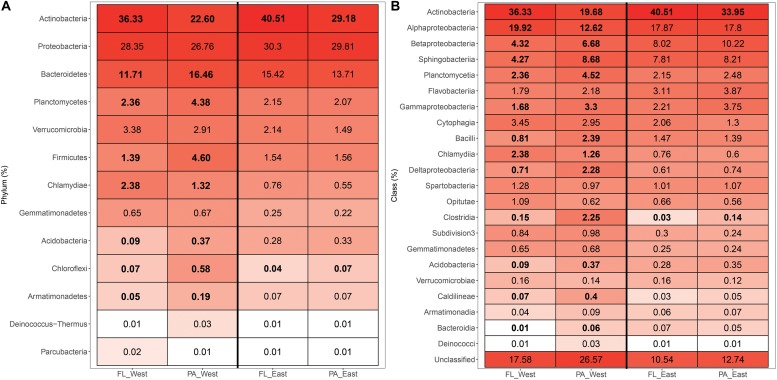
The taxonomic composition of the free-living (FL) and particle-attached (PA) bacterial communities at the phylum **(A)** and class **(B)** levels within the west lake Wuli and the east Lake Wuli. The digits in boldface indicate the phylotypes that were significantly different between the free-living (FL) and particle-attached (PA) bacterial communities.

A taxonomic comparison of the FL and PA bacterial communities on the basis cyanobacteria-free data was performed. Overall, the reads were classified and grouped under 16 and 18 phylum-level taxonomic groups for the FL and PA communities, respectively (Supplementary Figure S2). The predominant FL bacterial phyla were affiliated with Actinobacteria (38.56 ± 4.89%) and Proteobacteria (29.39 ± 3.40%), whereas the predominant PA bacterial phyla were Proteobacteria (31.23 ± 4.94%) and Actinobacteria (28.43 ± 10.91%). Within the west lake, the relative abundance of eight phyla significantly differed between FL and PA bacterial communities. Specifically, Actinobacteria was higher in FL fraction than in PA fraction (*P* = 0.006), whereas seven others were lower in the FL fraction than in the PA fraction (all *P* < 0.05) (Figure 8A). However, in the east lake, only two phyla significantly differed between the FL and PA bacterial communities: Actinobacteria and Chloroflexi (both *P* = 0.04), with Actinobacteria being higher in the FL communities and Chloroflexi being higher in the PA communities (Figure 8A).

At the finer taxonomic level, 30 class-level taxonomic groups were detected for the two bacterial communities (Supplementary Figure S2); both bacterial communities were dominated by Actinobacteria (38.56 ± 4.89% and 28.43 ± 10.91% in FL and PA, respectively) and Alphaproteobacteria (18.83 ± 3.66% and 14.64 ± 5.44% in FL and PA, respectively) (Figure 8B). In the west lake, 13 classes differed significantly between the FL and PA fractions (all *P* < 0.05), whereas in the east lake, only two classes differed between the fractions (both *P* < 0.05) (Figure 8B). At the genus level (Supplementary Figure S3), most of the OTUs could not be annotated to known genera, with only 156 and 164 members identified to belong to the FL and PA bacterial communities, respectively. The west lake had 47 genera, which significantly differed between the FL and PA fractions, whereas the east lake only had 21 genera that differed (all *P* < 0.05). Furthermore, in the west lake, some anaerobic bacteria, such as *Clostridium* and *Romboutsia*, were significantly enriched in the PA bacterial community relative to FL counterpart. However, in the east lake, the anaerobic bacteria of the FL and PA communities were comparable with each other.

### The Increasing Similarity of Functional Potentials Between the Free-Living and Particle-Attached Bacterial Communities From West Lake Wuli to East Lake Wuli

Based on the predicted bacterial Kyoto Encyclopedia of Genes and Genomes (KEGG) pathways, 276 KEGG orthology groups (KOs) were obtained in the FL and PA bacterial communities, among which 205 and 25 KOs significantly differed in the west lake and the east lake, respectively (all *P* < 0.05). In total, the KOs were clustered into 41 gene families, most of which belonged to membrane transport (14.24 and 12.62%), amino acid metabolism (12.63 and 12.19%), carbohydrate metabolism (12.14 and 12.40%), and signal transduction (7.19 and 8.16%) for FL and PA fractions, respectively. In the west lake, 68 KOs were significantly higher in the FL fraction than in the PA fraction, whereas 137 KOs were significantly higher in the PA fraction relative to the FL fraction. In the east lake, 11 KOs were significantly higher in the FL fraction than in the PA fraction, whereas 14 KOs were significantly higher in the PA fraction relative to the FL fraction.

**FIGURE 9:**
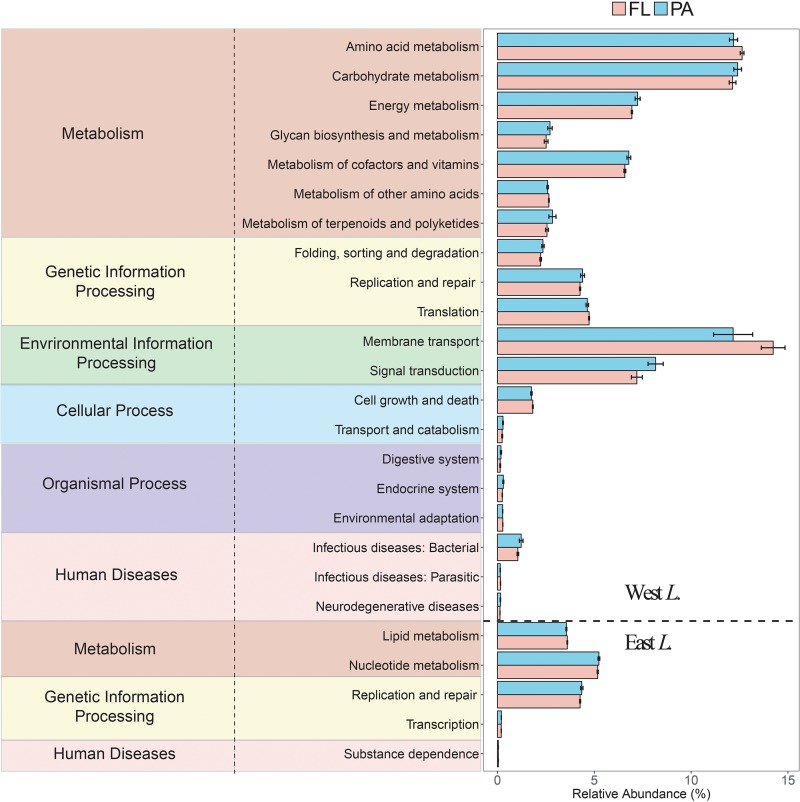
Functional profiles of the free-living (FL) and particle-attached (PA) bacterial communities within the west Lake Wuli and the east Lake Wuli.

In the west lake, only eight gene families showed significantly higher relative abundance in the FL fraction than in the PA fraction (all *P* < 0.05) (Figure 9), including membrane metabolism (mainly *ABC transporters* and *bacterial secretion system*), amino acid metabolism (mainly *glycine, serine, and threonine metabolism* and *arginine and proline metabolism*), metabolism of other amino acids (mainly *glutathione metabolism and phosphonate* and *phosphinate metabolism*). In contrast, 16 gene families were significantly higher in the PA fraction than in the FL fraction (all *P* < 0.05) (Figure 9), including carbohydrate metabolism (mainly *starch and sucrose metabolism* and *amino sugar and nucleotide sugar metabolism*), glycan biosynthesis and metabolism (mainly *lipopolysaccharide biosynthesis* and *other glycan degradation*), energy metabolism (mainly *methane metabolism* and *sulfur metabolism*), and signal transduction (mainly two-component systems). In the east lake, 14 gene families showed significantly higher relative abundance in the FL fraction than in PA fraction (all *P* < 0.05) (Figure 9). In contrast, 11 gene families were significantly higher in the PA fraction than in the FL fraction (all *P* < 0.05) (Figure 9).

## Discussion

Because FL bacteria and PA bacteria occupy different ecological niches, exploring their relationships sheds light on the nutrient cycles and major metabolic pathways in aquatic ecosystems. Some previous studies have demonstrated that FL and PA bacteria are significantly different from each other (Zhang et al., 2007; Crespo et al., 2013; Bachmann et al., 2018), whereas others argued that they are essentially the same and play only slightly different biogeochemical roles (Hollibaugh et al., 2000; Lamontagne and Holden, 2003). Thus, these conflicting observations prevent the understanding of functional processes of the aquatic ecosystems (Smith et al., 2013; Jain and Krishnan, 2017).

Our results showed that Lake Wuli could be divided into two significantly distinct aquatic regions: the east lake with slight eutrophication and the west lake with moderate eutrophication and hyper-eutrophication. The FL bacterial communities were less diverse than the PA fraction in the west lake, whereas they were as diverse as each other in the east lake. Furthermore, both compositional structures and metabolic functionalities of the FL and PA communities were significantly different in the west lake but become similar in the east lake. Thus, the FL and PA bacterial communities showed a convergent succession along the eutrophic gradient from the west lake to the east lake. Recently, an increasing body of evidence has shown that nutrient enrichment leads to biotic homogenization in aquatic habitats, including insects (Wengrat et al., 2018), macroinvertebrate (Zhang et al., 2019), and fish (Menezes et al., 2015), as well as phytoplankton (Monchamp et al., 2018). Thus, our results bear out our hypothesis that the relationships between the FL and PA bacterial communities are nutrient related.

### Suspended Particles Increase the Phylogenetic Overlap Between Free-Living and Particle-Attached Bacterial Communities

For decades, aquatic bacteria have been artificially categorized into two distinct lifestyles: FL and PA (Grossart, 2010). However, aquatic bacteria are capable of alternating between the FL and PA stages (Rösel and Grossart, 2012). A large body of compelling evidence demonstrated that there is an underestimated connectivity between FL and PA fractions (Tang et al., 2017). For instance, a study in experimental mesocosms showed that the PA bacteria were the important source for their FL counterparts (Riemann and Winding, 2001). A model-based method also quantitatively estimated their lifestyle alternation during a cyanobacterial bloom (Liu et al., 2019). Our study showed that TSS was positively associated with the OTUs exclusive to the FL fraction and was negatively associated with the OTUs exclusive to PA fraction. Moreover, TSS was also significantly positively correlated with the similarity between the FL and PA bacterial communities. Thus, TSS would play vital roles in controlling the relationships between the FL and PA bacterial communities (Dang and Lovell, 2016).

Firstly, increasing suspended particles promotes the desorption rate of PA bacteria and then separates them from the particles (Tang et al., 2017; Liu R. et al., 2018). Although this underlying mechanism has frequently been proposed, direct evidence is rarely presented. Our results provide firm evidence for this mechanism, as shown by the significant decrease of *two-component system* in PA bacterial communities from the west lake to the east lake. This signal transduction pathway controls bacterial cell attachment to the substrate surface, which facilitates biofilm formation (Dekkers et al., 1998; Hancock and Perego, 2004; Kulasekara et al., 2005). Notably, the gene expression of the *two-component system* is light dependent; therefore, light attenuation would markedly suppress expression of response regulator genes and consequently prevent the biofilm formation (Purcell et al., 2007). This is the case in the east lake, where higher TSS (than in the west lake) decreased light intensity in the water column promoting the transition from the PA to FL lifestyle.

Secondly, high suspended particles accompanied by intensive hydrodynamics also change the microenvironments of the particles surface. Owing to strong bacterial respiration, there are extensive low-oxygen and even anoxic microzones on the particle surface (Crump et al., 1999; Zhang et al., 2016). This condition was inferred from the significantly higher relative abundance of anaerobic bacteria in PA bacterial communities in the west lake than in the east lake, including genera such as *Clostridium* (Koo et al., 2018; Öner et al., 2018) and *Romboutsia* (Ricaboni et al., 2016; Maheux et al., 2017). High concentrations of suspended particles, with the help of hydrodynamics, cause intense collisions, which reduce the anoxic area on particle surface, so that the proportion of these anaerobic bacteria decreased in the east lake. Additionally, this ecological process was also verified by the change in energy metabolism of bacterial communities from the west lake to the east lake. In the west lake, bacterial metabolism was mainly restricted to obligatory anaerobic bacterial lineages, such as *methane metabolism* (Ettwig et al., 2010) and *sulfur metabolism* (Grein et al., 2013), which were significantly higher in the PA consortia than the FL consortia. In contrast, in the east lake, the relative abundance of such metabolisms was comparable in the FL and PA consortia. Thus, suspended particles with the hydrodynamics increase the microenvironment similarity of particles and their surrounding water in terms of oxygen availability.

### Carbon Promotes the Similarity of Functional Metabolisms Between Free-Living and Particle-Attached Bacterial Communities

Our PICRUSt analysis predicted that in the west lake, the functional metabolisms were significantly different between FL and PA bacterial communities, which was consistent with previous observations in different aquatic habitats (Ghiglione et al., 2010; Lapoussière et al., 2012). In the present study, the PA fraction had more KOs than did the FL fraction (137 vs. 68), which was congruent with the consensus that the PA fraction is more highly active than the FL fraction (Milici et al., 2016; Liu R. et al., 2018). In the PA fraction, the following functional gene categories were significantly enriched, namely, those relevant to *methane metabolism*, *nitrogen metabolism*, *sulfur metabolism*, and *carbon fixation pathways in prokaryotes*. In contrast, in the FL fraction, only *carbon fixation in photosynthetic organisms* was enriched. These findings indicated that the PA consortia may contribute more in biogeochemical cycle in aquatic ecosystems than does the FL consortia (Rösel and Grossart, 2012). Indeed, PA bacteria have larger and more variable genomes than do FL bacteria, which enable a variety of metabolic and regulatory capabilities equipping cells to take advantage of organic materials (Smith et al., 2013; Luo and Moran, 2015). Moreover, the gene categories involved in *carbohydrate metabolism* and *glycan biosynthesis and metabolism* are significantly richer in the PA fraction, whereas the FL fraction is significantly higher in *amino acid metabolism* and *metabolism of other amino acids*. This finding indicates the distinct preferences for organic materials of the FL and PA communities, which agrees with a previous study showing that FL and PA consortia favor quite different environmental conditions (Xu et al., 2018).

Similar to the convergent succession of the bacterial communities, there was a significant decrease of metabolic differences between the FL and PA fractions from the west lake to the east lake. We speculate that this is due to the carbon source, based on the strong associations of the similarities between these two fractions with COD, DOC, and Chl-*a*. In eutrophic lakes where nitrogen and phosphorus have been adequately provided, carbon appears to be the limiting resource for heterotrophic bacteria (Tang et al., 2017). Especially in the west lake, the major carbon species were recalcitrantly allochthonous from the riverine discharge, consisting mainly of higher plants, soil humics, and phytodetritus (Hollibaugh et al., 2000). A recent study also revealed that approximately 60% of water-soluble organic matters are humic-like components in Lake Wuli (Wang et al., 2018). Thus, the PA bacteria owing to their closer distance to particles and higher metabolic activities are more competitive in utilizing carbon source than are the FL bacteria (Luo and Moran, 2015; Liu et al., 2019). The FL bacteria may supplement their metabolism by using amino acids as their carbon sources, as indicated by their enriched gene families involved in amino acids metabolism (Bertilsson et al., 2007). In the east lake, the algae blooms induced by the moderately eutrophic and hyper-eutrophic condition released biopolymers (mainly polysaccharides) into the surrounding water, alleviating the restriction of carbon sources to FL bacteria (Villacorte et al., 2015; Liu C. et al., 2018). In this regard, the available organic matter released from the massive algal blooms provided a similar environment on and off particles (Liu et al., 2019) and consequently promoted the similarity of functional metabolisms between the FL and PA bacterial communities.

## Conclusion

In this study, the relationships between the FL and PA bacterial communities along the eutrophic gradient were investigated in Lake Wuli in China. In terms of diversity, taxonomic structure, and functional metabolism, the FL and PA bacterial communities were significantly different from each other in the west lake, whereas they were comparable with each other in the east lake. This convergent succession between the FL and PA bacterial communities was controlled by suspended particles and carbon resource. Thus, our results imply that the connectivity between the FL and PA bacterial communities is nutrient related.

## Data Availability Statement

The sequencing data generated by this study can be found in the BIG Nation Genomics Data Center (https://bigd.big.ac.cn/search?dbId=gsa&q=CRA002048).

## Author Contributions

YH and GG conceived and designed the experiments. GX and XJ performed sample collection. KS and XT analyzed the data. YH wrote the manuscript. All authors have read and approved the final manuscript.

## Conflict of Interest

The authors declare that the research was conducted in the absence of any commercial or financial relationships that could be construed as a potential conflict of interest.

## Footnotes

1 http://www.r-project.org

## Supplementary Material

FIGURE S1 The rarefaction curve of the FL and PA bacterial communities.

FIGURE S2 The number of OTUs of the FL and PA bacterial communities at the distinct levels within the west Lake Wuli and the east Lake Wuli.

FIGURE S3 The relative abundance of the FL and PA bacterial communities at the genus levels within the west Lake Wuli and the east Lake Wuli.
