# Supplementary figures and images for: The Relationships Between the Free-Living and Particle-Attached Bacterial Communities in Response to Elevated Eutrophication
Source: Front Microbiol. 2020 Mar 25;11:423. doi: 10.3389/fmicb.2020.00423 (PMC7109266; doi:10.3389/fmicb.2020.00423)

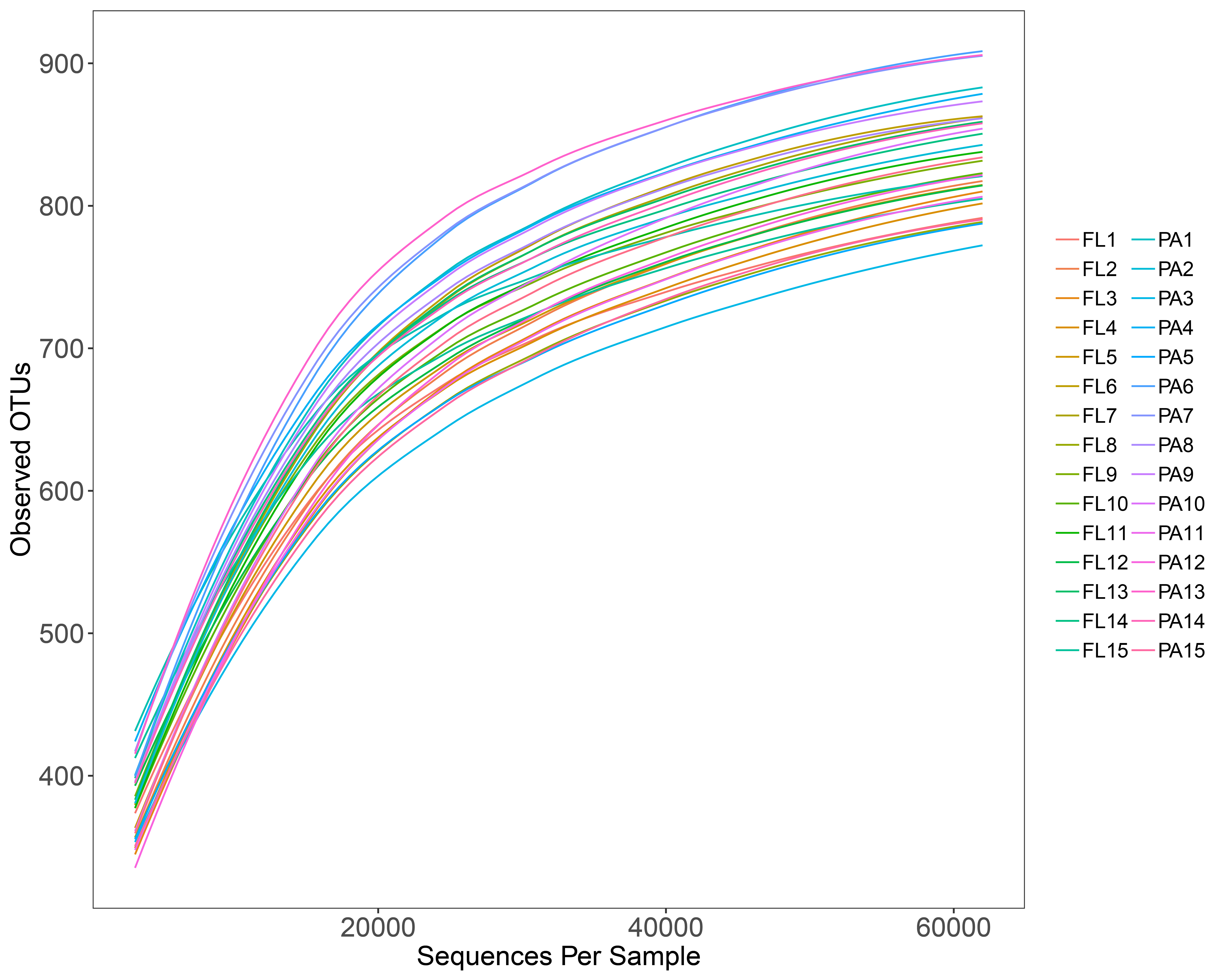

Supplement: FIGURE S1 — The rarefaction curve of the FL and PA bacterial communities. [file Image_1.JPEG]

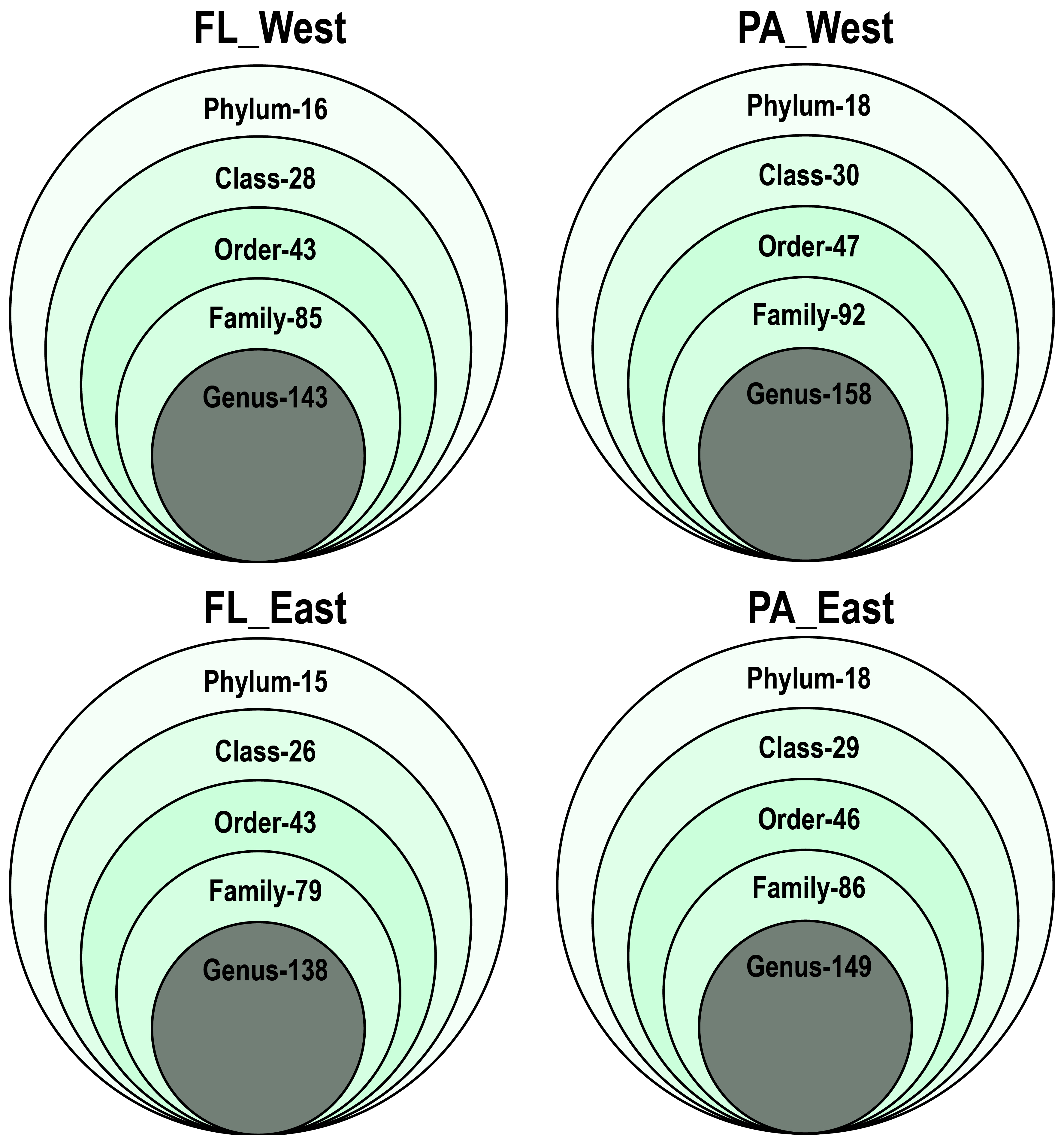

Supplement: FIGURE S2 — The number of OTUs of the FL and PA bacterial communities at the distinct levels within the west Lake Wuli and the east Lake Wuli. [file Image_2.JPEG]

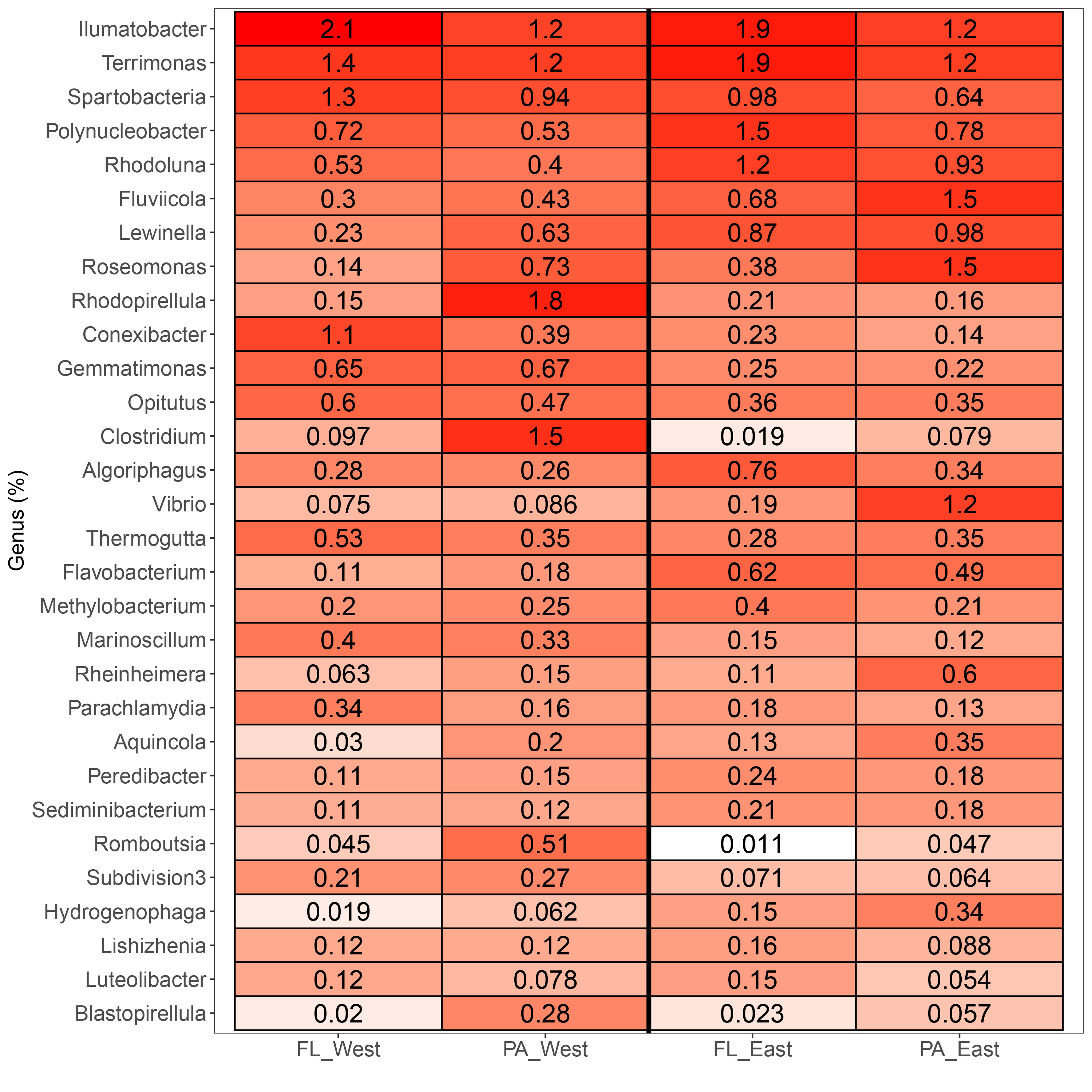

Supplement: FIGURE S3 — The relative abundance of the FL and PA bacterial communities at the genus levels within the west Lake Wuli and the east Lake Wuli. [file Image_3.JPEG]
